# Nutrient Loading and Viral Memory Drive Accumulation of Restriction Modification Systems in Bloom-Forming Cyanobacteria

**DOI:** 10.1128/mBio.00873-21

**Published:** 2021-06-01

**Authors:** Spiridon E. Papoulis, Steven W. Wilhelm, David Talmy, Erik R. Zinser

**Affiliations:** a Department of Microbiology, University of Tennessee, Knoxville, Tennessee, USA; University of Georgia

**Keywords:** *Microcystis*, harmful algal blooms, viral defense, eutrophication

## Abstract

The mechanisms driving cyanobacterial harmful algal blooms (HABs) like those caused by Microcystis aeruginosa remain elusive, but improved defense against viral predation has been implicated for success in eutrophic environments. Our genus-level analyses of 139,023 genomes revealed that HAB-forming cyanobacteria carry vastly more restriction modification systems per genome (RMPG) than nearly all other prokaryotic genera, suggesting that viral defense is a cornerstone of their ecological success. In contrast, picocyanobacteria that numerically dominate nutrient-poor systems have the fewest RMPG within the phylum *Cyanobacteria*. We used classic resource competition models to explore the hypothesis that nutrient enrichments drive ecological selection for high RMPG due to increased host-phage contact rate. These classic models, agnostic to the mechanism of defense, explain how nutrient loading can select for increased RMPG but, importantly, fail to explain the extreme accumulation of these defense systems. However, extreme accumulation of RMPG can be achieved in a novel “memory” model that accounts for a unique activity of restriction modification systems: the accidental methylation of viral DNA by the methyltransferase. The methylated virus “remembers” the RM defenses of its former host and can evade these defenses if they are present in the next host. This viral memory leads to continual RM system devaluation; RMs accumulate extensively because the benefit of each addition is diminished. Our modeling leads to the hypothesis that nutrient loading and virion methylation drive the extreme accumulation of RMPG in HAB-forming cyanobacteria. Finally, our models suggest that hosts with different RMPG values can coexist when hosts have unique sets of RM systems.

## INTRODUCTION

The ecological success of harmful algal bloom (HAB)-forming species is not well understood despite the global threats of HABs (1). HAB-forming cyanobacteria, such as *Microcystis* spp., can produce toxins that threaten the health and safety of humans and animals interacting with contaminated water and can often damage local economies. It is well established that anthropogenic nutrient loading facilitates the proliferation of these harmful cyanobacteria, leading to the accumulation of dense phytoplankton biomass, as often witnessed in freshwater systems such as Lake Erie (North America) or Lake Taihu (China) (2, 3). Multiple studies have investigated the complex interactions between genetic potential, bottom-up (e.g., nutrient loading) and top-down (2, 4, 5) controls that lead to the ecological success of HAB-forming cyanobacteria. However, we still lack mechanistic frameworks that incorporate these different selective pressures together to elucidate the drivers of ecology and evolution of these domestic pests.

Viruses (bacteriophages or phages) are powerful and ubiquitous evolutionary drivers in the prokaryotic world (6). The lysis of microbial cells contributes to biogeochemical recycling via a process known as the “viral shunt” (7) and selects for genotypes resistant to viral infection. Antiviral innovations fall into two general classes: those that prevent virus adsorption at the cell envelope, e.g., through mutation of the virus receptor (8–10), and those that establish within the cytoplasm the ability to destroy the virus or kill the infected cell. Cytoplasmic defenses are widespread in prokaryotes (11, 12) and include CRISPR (13), argonauts (14), toxin-antitoxin systems (15), abortive infection (16), and BREX (17). While many of these cytoplasmic defenses have only recently been discovered, restriction modification (RM) systems have been investigated since the 1950s (18, 19).

RM systems galvanized the molecular biology revolution through their ability to cleave double-stranded DNA (dsDNA) at sequence-specific motifs. When expressed *in vivo*, the endonuclease (restriction enzyme) activity of the RM system can protect a potential host cell from dsDNA viruses that contain the specific sequences recognized by the RM. Individual RMs can reduce rates of infection by 2 to 6 orders of magnitude (20). Because of this antiviral effect, RMs can be thought of as innate immune systems whose targets are predetermined by the specified recognition motifs of the endonucleases. This contrasts with the “adaptive immunity” conferred by CRISPR-Cas systems that use information gathered from prior infections to provide targets for DNA cleavage (13).

Because the DNA motifs targeted by the endonuclease of RMs are short, usually spanning 6 bases (but can range from 4 to 14) (21), they are usually found in multiple sites of the phage DNA; however, they also often occur within the host’s much larger genome. To protect the cell’s genome from cleavage at these motifs, most RMs also provide DNA methyltransferase activity that methylates residues within the same target motif as the endonuclease. For type I to III RMs (see below), the endonuclease activity is specific for unmethylated DNA. Thus, the role of the methyltransferase is to block the endonuclease from cleaving host DNA, while leaving it free to attack incoming, unmethylated viral DNA. One important drawback to this defense system is that any viral DNA that escapes endonuclease attack long enough will be “immunized” by the methyltransferase (18–20, 22–25). Methylated viral progeny released from the cell will be protected from endonuclease activity if infecting a new cell with the same RM defense. Knowing the recognition sequence is critical in determining whether a methylated virus will be able to evade a host endonuclease. For example, viruses methylated at the motif GATATC would not be resistant to an endonuclease that targets GAATTC.

RMs fall into one of several classes based on protein structure and DNA target. In type II RMs, endonuclease and methyltransferase activities are in separate proteins that recognize DNA independently. Type I and III RMs involve separate proteins that complex together with or without a specificity unit, respectively. Types IIB, IIG, and IIH (collectively referred to as type IIG here) are single polypeptides with both activities covalently linked. Finally, type IV RMs are single endonucleases that cleave methylated, rather than unmethylated, DNA (26).

Previously, Microcystis aeruginosa was found to have the greatest fraction of its genome devoted to antiviral defenses relative to all other prokaryotes, suggesting that defense against viral predation is a cornerstone of its ecological success as a bloom-forming cyanobacterium (27). In the present study, we tested this hypothesis in an RM-specific context to determine whether HABs are unique in their capacity to defend against foreign DNAs, such as viruses, relative to the rest of the prokaryotic world. Moreover, because of the consistency in the DNA cutting mechanism of RMs, we used mathematical models to explore the causal effect of eutrophication, a bottom-up control, on the selective pressures acting on the defense against viral predation, a top-down control. We begin with a bioinformatic assessment of the distribution of RM systems in prokaryotes. We go on to highlight interesting ecological patterns in RM systems per genome (RMPG) in diverse cyanobacteria. Finally, we introduce three mathematical population models used to evaluate ecological and molecular mechanisms of RM selection in contrasting nutrient conditions.

## RESULTS

### Restriction modification distributions in prokaryotes.

**FIG 1:**
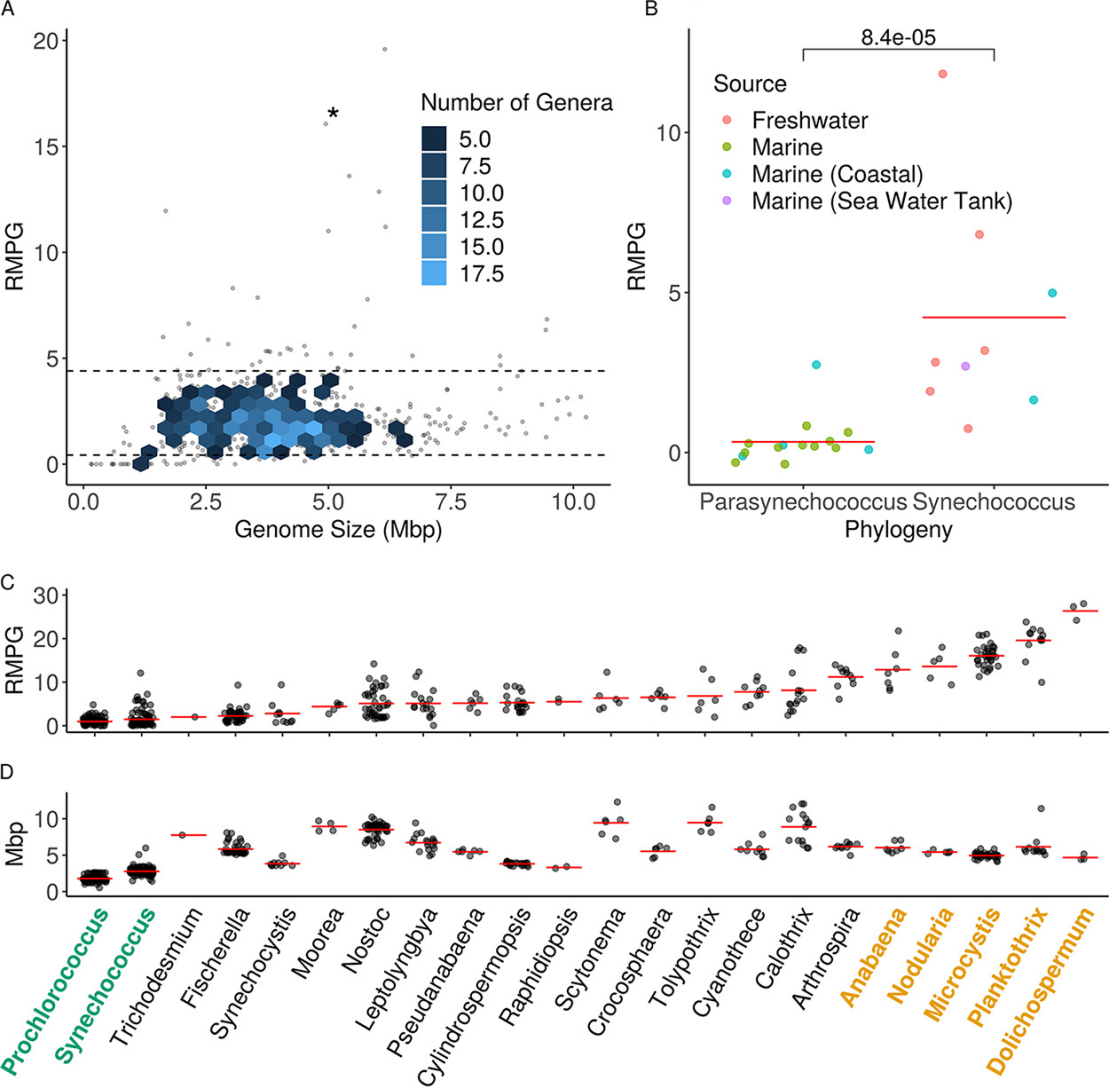
Distributions of RM systems. (A) 95% quantile (≥4.4 RMPG) of the RM distribution from all prokaryotes. Error bars represent 95% confidence intervals of the genus mean, and data points represent five or more isolates. Genera were dropped if the 95% confidence interval fell below the 95th quantile. (B) Subset of *Synechococcus* genomes parsed according to Coutinho et al. (37), into either *Parasynechococcus*, which is closely related to *Prochlorococcus*, or (remaining as) *Synechococcus*. Genomes were color coordinated to their isolation environment. The Wilcoxon rank sum *P* value is 8.4 × 10^−5^. (C and D) *Cyanobacteria* phylum, showing complete RMPG (C), and genome size (D), ordered by RMPG. Colored genera are from oligotrophic (green) or eutrophic (yellow) environments. Red bars indicate the genus mean, while data points indicate individual isolates. All genera have five or more isolates except for *Raphidiopsis* (*n* = 2), *Trichodesmium* (*n* = 1), and *Dolichospermum* (*n* = 3).

We developed a custom pipeline to quantify the RMPG among the 139,023 high-quality bacterial and archaeal genomes available in the RefSeq database (see Data Set S1 in the supplemental material). To mitigate the disproportionate contribution by some taxa (e.g., genomes from overrepresented genera such as *Shewanella* and Escherichia) to the prokaryote mean, we aggregated data at the genus level (see Data Set S1). For the 2,522 bacterial and archaeal genera analyzed, a mean value of 2.475 and a median of 2.0 RMPG were observed, with 5th and 95th quantiles of 0 and 6 RMPG, respectively. Further restriction of the data set to include only genera with 5 or more sequenced genomes yielded mean, median, and 5th and 95th percent quantiles of 2.17, 1.91, 0.427, and 4.40 RMPG, respectively (Fig. 1A).

**FIG 2:**
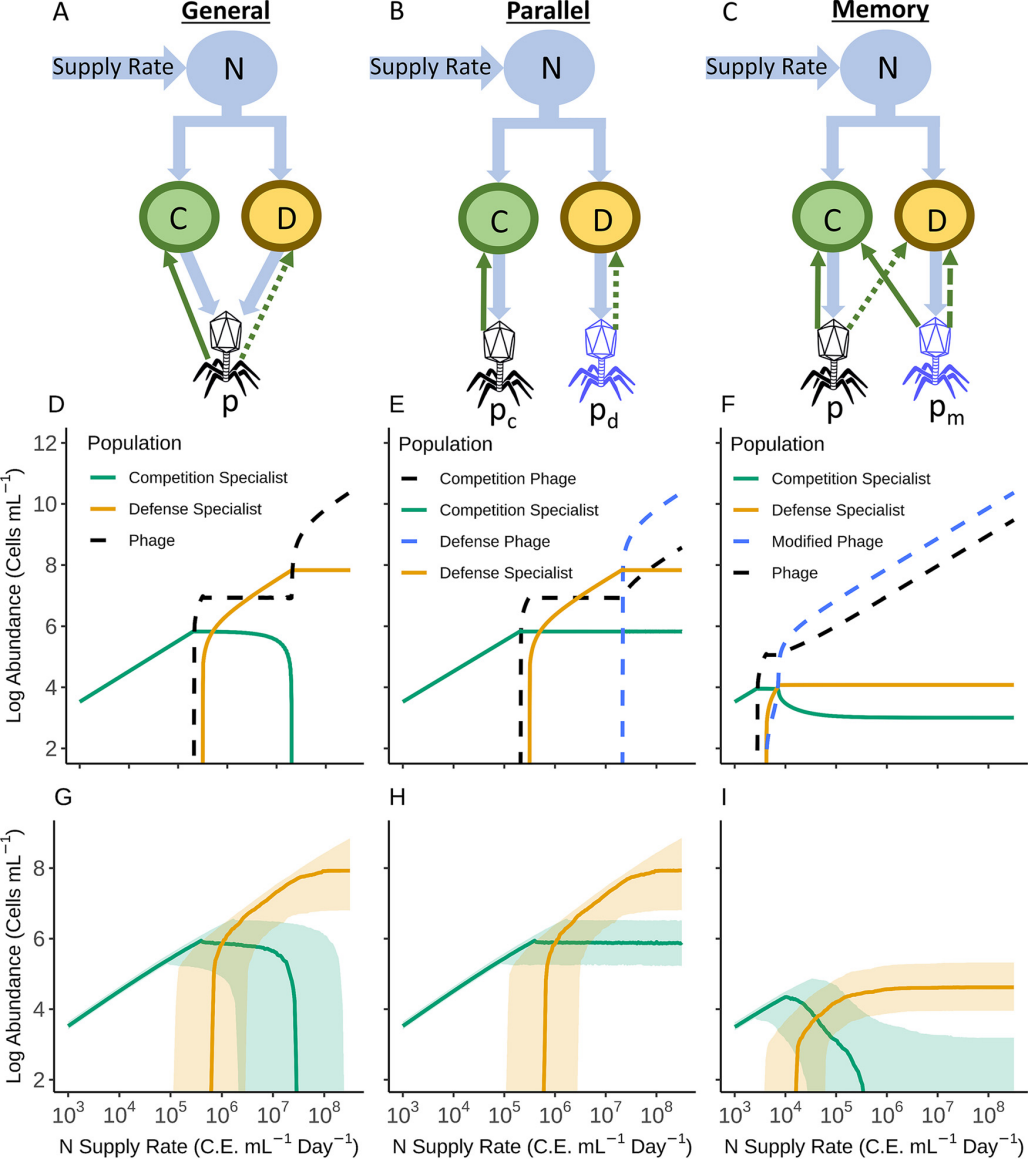
General, parallel, and memory virus-host interaction models. Figure columns correspond to general (A, D, and G), parallel (B, E, and H), and memory interaction (C, F, and I). Model structures (A to C) show the mass transfer from resource (N), to competing competition (C) and defense (D) specialist host populations, and finally into phage (p). Phage subscripts denote competition (c) or defense (d) specialist hosts (E) or phage modified by methylation (m) (F). Competition and defense specialists have 1 or 2 RMPG values, respectively. Solid green arrows represent high infection rates, dashed lines represent intermediate infection rates, while dotted lines represent low infection rates. (D to F) Steady-state abundance of each prokaryotic and viral population across a wide range of resource supply rates (*S* in equation 1). Solid gold and green lines are the defense and competition specialists, respectively. Dashed black lines show the abundance of phage, while the dashed blue line shows the abundance of the competition phage in the parallel model or the modified phage in the memory model. (G to I) Variation in steady-state values for the competition and defense specialists from 1,000 simulations with parameters drawn from realistic distributions (see Materials and Methods). Solid lines indicate the median value of each population, while the shaded regions show the 75th and 25th quantiles. C.E., cellular equivalents. For parameter values, see Table S1 in the supplemental material.

**FIG 3:**
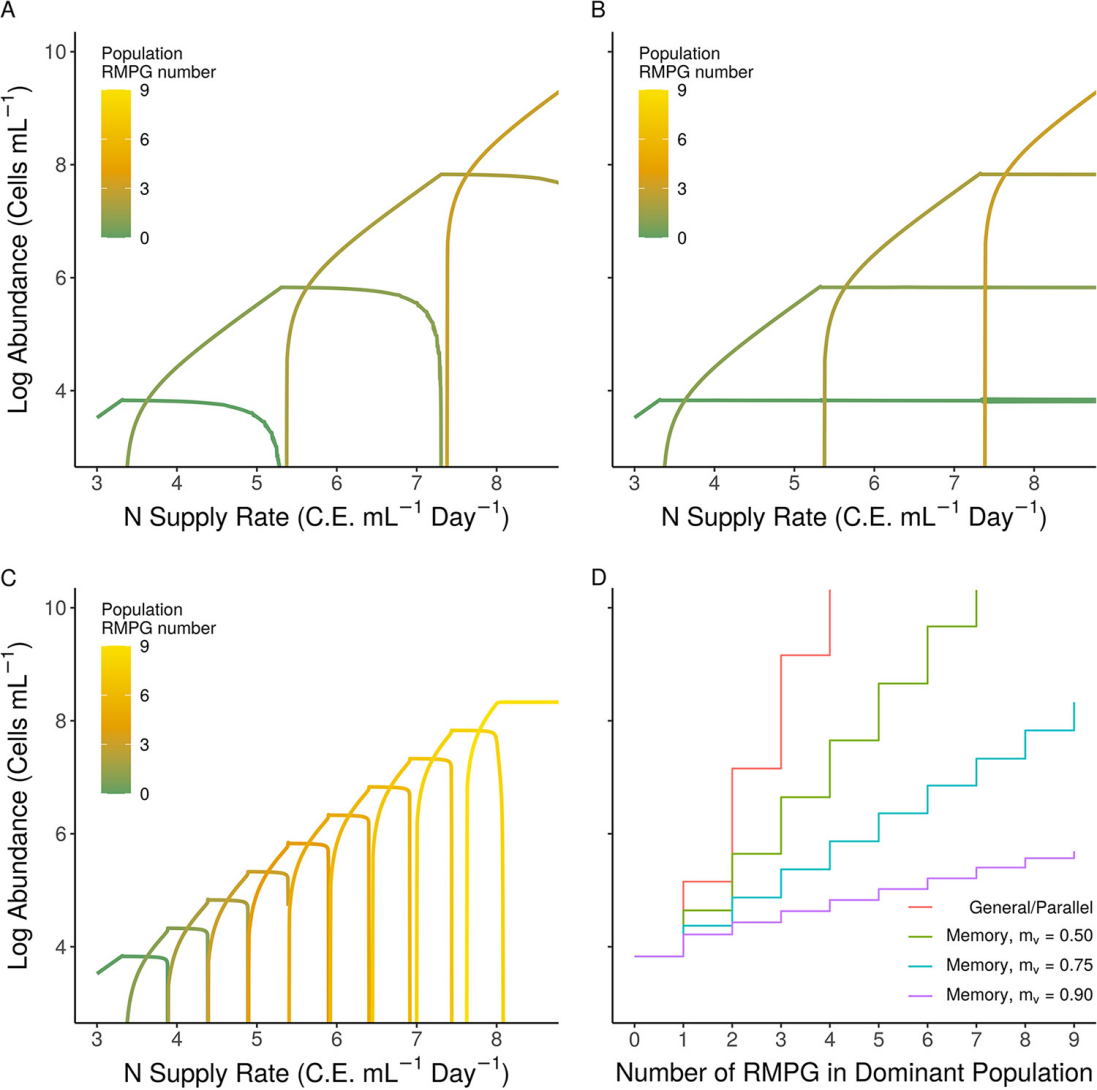
Abundance scaling with increasing defense types in general, parallel and memory models. (A, B, and C) Steady-state abundances of populations carrying different numbers of RM systems. For simplicity, we assumed the cost and resistance of each additional RM system are identical. (C) Virion methylation (*m_v_*) is set to 0.75, and RMs are in a “subset” arrangement (see the text for further description). (D) Total abundance of each community plotted against the number of RM systems in the dominant subpopulation. General and parallel models are identical, while the scaling of the memory model depends on the partial resistance conferred from the degree virions are methylated. C.E., cellular equivalents. For other parameters, see Table S1.

10.1128/mBio.00873-21.8 TABLE S1 Parameter values. Values used for simulations in Fig. 2, 3, and 4. Please see Materials and Methods for a detailed description of the equations. Download Table S1, DOCX file, 0.01 MB. Copyright © 2021 Papoulis et al. 2021 Papoulis et al. https://creativecommons.org/licenses/by/4.0/ This content is distributed under the terms of the Creative Commons Attribution 4.0 International license.

10.1128/mBio.00873-21.9 DATA SET S1 Table 1 shows the RefSeq assemblies used in this study. RefSeq metadata, including .ftp locations of source materials, in .csv format are shown. Table 2 shows the RM counts aggregated at the genus level. CSV file of organism genome and RM count (i.e., RMPG) data aggregated at the genus level, where all numeric columns are averages, except for those column names delimited with “_std”, which are standard deviations. Fields include taxonomic information (genus, phyla), genome information (num_isolates, bp, NumContigs), and RM counts. RM counts are distinguished by codes, where r = restriction enzyme, m = methyltransferase, and the number indicates the type. Total RM counts are a summation of rmT1, rmT2, rmT3, T4, and T2G_posthoc, where total RM without putative type IIG RM systems (see Fig. S2) are a summation of rmT1, rmT2, rmT3, T4 and T2G. Table 3 presents rebase “Gold Standard” nonputative methyltransferases and endonucleases. Proteins used to retrieve HMM profiles, in fasta format. Table 4 shows RM Pfam value found in Rebase “Gold Standard” proteins. A list of HMMs was used to identify RM as a text file. This file can be used to retrieve HMMs out of Pfam using hmmfetch. Table 5 shows Pfam values that covary with false positives. A list of HMMs used to identify common false positives as a text file (see SI methods). This file can be used to retrieve HMMs out of Pfam using hmmfetch. Table 6 shows BLAST exception proteins. A subset of data from Table 3 that do not have Pfam values detailed in Table 4, in fasta format, is shown. Download Data Set S1, XLSX file, 11.2 MB. Copyright © 2021 Papoulis et al. 2021 Papoulis et al. https://creativecommons.org/licenses/by/4.0/ This content is distributed under the terms of the Creative Commons Attribution 4.0 International license.

10.1128/mBio.00873-21.2 FIG S2 Distribution of restriction modification systems in prokaryotic organisms without type IIG RM found with HHblits. (A) Mean RMPG plotted against mean genome size in prokaryotic genera. Data points represent the mean of five or more isolates; hexagons are rendered when there are five or more data points. Mean RMPG and 95% confidence interval = 1.81 ± 0.09; median RMPG = 1.59. Dotted horizontal lines denote the 5th and 95th percent quantile cutoffs. An asterisk is adjacent to the mean RMPG data point for *Microcystis.* (B) Subset of *Synechococcus* genomes parsed according to Coutinho et al. (37) into either *Parasynechococcus*, which is closely related to *Prochlorococcus*, or (remaining as) *Synechococcus*. Genomes were color coordinated to their isolation environment. The Wilcoxon rank sum *P* value is 3.2 × 10^−4^. (C and D) *Cyanobacteria* phylum, showing complete RMs per genome (C) and genome size (D). Genera are in ascending rank order by genome size. Red bars indicate genus mean, while data points are individual isolates. All genera have five or more isolates except for *Raphidiopsis* (*n* = 2), *Trichodesmium* (*n* = 1), and *Dolichospermum* (*n* = 3). Download FIG S2, TIF file, 2.8 MB. Copyright © 2021 Papoulis et al. 2021 Papoulis et al. https://creativecommons.org/licenses/by/4.0/ This content is distributed under the terms of the Creative Commons Attribution 4.0 International license.

The low-RMPG genera (0 to 5th percent quantile, <0.427 RMPG) included several organisms that are exclusively intracellular or have a large intracellular component to their lifestyle, such as *Wolbachia* and *Rickettsia* (28) (see Fig. S1A). Given that a strict intracellular lifestyle should limit contact with infectious viruses and thus reduce the pressure to maintain viral defense, it was not surprising to find these genera in the low-RM category. The high-RMPG genera (95th to 100th percent quantile, >4.40 RMPG) included *Microcystis*, as well as the heterotrophic genera *Helicobacter* and *Neisseria*, both noted previously for their high number of RMPG (29, 30) (see Fig. S1B).

10.1128/mBio.00873-21.1 FIG S1 Extremes of the RMPG distribution. (A) Genera represent the 5% quantile (≤0.427 RM) of the prokaryotic RMPG distribution. The mean number of complete RMPG plotted against mean genome size in prokaryotic genera is shown. Data points represent the mean of five or more isolates; hexagons are rendered when there are five or more data points. Genera were removed from this plot of the 95% confidence interval crossed the 5% quantile threshold. (B) 95% quantile (≥4.4 RM) of the RM distribution from all prokaryotes. Error bars represent 95% confidence intervals of the genus mean, and data points represent five or more isolates. Genera were dropped if the 95% confidence interval fell below the 95th quantile. Download FIG S1, TIF file, 3.0 MB. Copyright © 2021 Papoulis et al. 2021 Papoulis et al. https://creativecommons.org/licenses/by/4.0/ This content is distributed under the terms of the Creative Commons Attribution 4.0 International license.

Previous studies reported a correlation between genome size and RMPG (31–33). To revisit these analyses, we performed both linear and negative binomial regressions on the mean RMPG of the genera with five or more sequenced genomes. Both regressions give the same result: genome size is a poor predictor of the number of RMs in prokaryotes as it can explain no more than ∼2% of the variation (linear: estimate = 0.145, *R*^2^ = 0.0217, *P* = 4.93 × 10^−5^; negative binomial: estimate = 0.06401, McFadden Pseudo *R*^2^ = 7.02 × 10^−3^, *P* = 1.07 × 10^−5^). Moreover, while these trends are statistically significant, the estimates from each regression suggest there would need to be a large increase in genome size for there to be an impact in RM count if genome size is the sole predictive indicator. For example, an organism with an initial genome size of 2 Mbp would need to expand its genome by an additional 6.90 or 6.50 Mbp according to linear or negative binomial regressions, respectively, to gain one RM system. However, for small genomes within the range of 0.5 to 2.5 Mbp, we observed a more pronounced scaling of RMPG as a function of genome size, a trend that is consistent with earlier studies (32, 33).

### Patterns of RM defense in cyanobacteria.

Noting an absence of strong indicators for RMPG scaling across all prokaryotes, the analysis was subsequently restricted to the phylum *Cyanobacteria*, whose members include *Microcystis* and other genera for which their ecology is well known. This restriction provided an opportunity to relate RMPG to organism lifestyle in addition to genome size.

The range of RMPG among the cyanobacterial genera was nearly as extensive as for all prokaryotes (Fig. 1C) and did not trend with genome size (Fig. 1D). The high end of the cyanobacterial RMPG distribution was dominated by the HAB-forming freshwater genera *Microcystis*, *Planktothrix*, *Nodularia*, *Dolichospermum*, and *Anabaena* (34) (Fig. 1C). This signal was robust even with more-stringent annotation calls (see Materials and Methods; see also Fig. S2 in the supplemental material), suggesting a strong association between bloom formation and RM abundance.

The bottom of the RM distribution is occupied by unicellular picocyanobacteria, *Prochlorococcus* and *Synechococcus* spp. (Fig. 1C), with many *Prochlorococcus* genomes lacking RMs altogether. *Prochlorococcus* numerically dominates the low nutrient (oligotrophic) oceans and, while peaking at about 10^5^ cells ml^−1^, is the most abundant photosynthetic organism on Earth (35). *Synechococcus* is a picocyanobacterium with a broad habitat range: while some genotypes contribute significantly to the marine oligotrophic phytoplankton community, other genotypes can be found at high abundance in nutrient-rich coastal environments or in freshwater systems (36). Given these varied ecologies, intragenus trends in RMPG were assessed using the classification of Coutinho et al. that phylogenetically distinguishes the open ocean genotypes (e.g., *Parasynechococcus*) from other, typically freshwater, genotypes (e.g., *Synechococcus*) (37). Interestingly, genomes of *Parasynechococcus*, which are more closely related to the oligotrophic specialist *Prochlorococcus*, showed statistically fewer RMPG (*P* = 8.4 × 10^−5^, Wilcoxon rank sum; Fig. 1B). Moreover, we found that isolates from open ocean marine systems skew toward the bottom of the *Synechococcus* RMPG distribution.

The observed correlation between extremes in RMPG and cell densities of the dominant genus suggested that cell density, as a function of nutrient availability, is an important driver of RM acquisition or loss in the cyanobacterial lineage. The high end of the RMPG distribution was dominated by HAB-forming cyanobacteria, whose blooms are largely attributed to eutrophication of water bodies from farm runoff carrying fertilizer, flooding the system with nitrogen and phosphate which promote life at high density (38). The low end was dominated by oligotrophic picocyanobacteria that are deprived of nutrients due to temperature stratification of the deep ocean and large geographic distances from coastal inputs. Given these relationships, we hypothesized that the trade-off between defense and nutrient utilization plays a central role in the evolution of RM defenses and that loss or gain is tied to nutrient availability.

### Evaluating mechanisms of RM selection.

Motivated by the observation that high nutrient input is associated with the occurrence of HAB-forming cyanobacteria with high RMPG (Fig. 1), we built upon existing ecological models (39–43) to evaluate competition in contrasting nutrient regimes. We made no attempt to encapsulate genotypic or phenotypic diversity, other than through RM-associated differences in ability to protect against viruses, which in all cases we associated with a cost to nutrient utilization ability. “Competition” specialists (low RMPG) were competed with “defense” specialists (high RMPG) in “general” and “parallel” models that encapsulate interactions within nested and parallel infection networks, respectively (see Materials and Methods for further details). We hypothesized that a low nutrient supply places strong pressure on cells to acquire nutrients, forgoing investment in RM defenses even at the cost of viral attack. We hypothesized that nutrient enrichments alleviate pressure to acquire resources and drive ecological selection for RM systems due to an increasing host-phage contact rate.

The two ecological models (Fig. 2A and B) capture the governing role of nutrient enrichment on competitive outcomes while remaining agnostic to the mechanism of defense. We developed a third, “memory” model (Fig. 2C) in which methylation of virions by host methyltransferases is captured. The parallel and general models serve as controls, allowing us to isolate the unique effects of viral methylation on investment in RM systems in contrasting nutrient conditions.

### Phage-host interaction models lacking a memory function incompletely explain RM accumulation.

We began by evaluating competition between an organism carrying 1 RMPG (competition specialist) with a newly emerged 2-RMPG subpopulation after a gain of function event (defense specialist) (Fig. 2). Outcomes were qualitatively similar when a 1-RMPG subpopulation emerged from one with 0 RMPG (data not shown). Outcomes for both general and parallel models could be binned generally by nutrient inflow as low, midrange, and high. In the “general model,” where a single generalist phage can infect both hosts, a low nutrient supply established a steady-state monoculture of the competition specialist, as both the defense specialist and the phage were eliminated from the system (Fig. 2D and G). A midrange nutrient supply led to coexisting steady-state populations of competition and defense specialists. Within this range, the defense specialist cell density scales with nutrient supply rate, whereas the competition specialist density is held in check by the virus. At the highest nutrient supply examined, the system enters a new state where the competition specialist is driven to extinction, and the defense specialist scales with nutrient supply rate until its density is held in check by phage, the latter scaling with nutrient input rate.

In the “parallel model,” outcomes for the competition and defense specialists at low to mid-range nutrient inflows are like the general model (Fig. 2E and H). In contrast to the general model, however, the parallel model predicts stable coexistence of defense and competition specialists at high nutrient supply. Both models thus indicate that carrying two RMs at a high nutrient supply confers selective advantages at high cell densities over hosts with only one.

Models were next examined for their ability to explain the escalation of RM defenses such as observed in *Microcystis* (RMPG ≈ 16; Fig. 1C). When present in the same cell, RMs targeting different DNA sequences confer multiplicative effects on viral defense (22, 44). Assuming an equal number of restriction sites per viral genome and identical efficiencies among the endonucleases per restriction site, if a single RM system confers a moderate infection reduction of 100-fold (20), then the addition of a second RM system would reduce infections 10,000-fold.

Redundance of RM systems becomes evident in both control models when hosts with *n* = 1, 2, 3 RMPG compete for nutrients (Fig. 3A and B). Assuming a single RM system has a modest reduction in infections of 100-fold (20) (resistance gained per endonuclease in our model [*r_e_*] = 100), three RMPG is sufficient to fully protect against viruses over a range of cell densities up to roughly 10^10^ cells ml^−1^. Hosts with >3 RMPG are not selected, even for modest assumed costs of carrying an RM (Fig. 3A and B). The selective basis for genera such as *Microcystis*, which has maximal cell densities ∼8 × 10^8^ ml^−1^ (45), to carry more than 16 RMPG is thus poorly reconciled with the efficiency of endonucleases by these models. Clearly, our control models are unable to scale RMPG between ecological extremes when *Prochlorococcus* genomes typically carry 0 to 1 RMPG and have maximal cell densities of ∼2 × 10^5^ in the oligotrophic ocean (46).

### Viral methylation drives RM escalation and de-escalation.

Given the poor explanatory power that the two initial models provided for the observed escalation of RM defenses in *Microcystis* and other HAB-forming cyanobacteria, a new model that incorporates the viral memory conferred by the methyltransferase component of the host’s RM was developed (Fig. 2C). In this model, the (rare) surviving progeny phage of RM-expressing hosts develop immunity to that RM via methylation of its genome during infection and thus can infect both the defense and competition hosts at equal rates if the viral genome is fully methylated. We further explored relaxing this assumption by incorporating a “virion methylation” parameter (*m_v_*) which allows for virions to be hypomethylated. Virion methylation is a new parameter relative to previous modeling of RMs (24, 47, 48) and toggles the degree that progeny virions adopt the hosts methylation state. While some classic works suggest that most if not all virions are methylated in each viral burst (*m_v_* ≈ 1) (23, 25), we felt the possibility of hypomethylation of virions should not go overlooked since these works focused almost exclusively on type I RMs, with the exception of EcoRI and EcoRII which are type II RMs. Indeed, assuming plasmid conjugal transfer efficiency in *Nostoc* PCC 7120 is a proxy for resistance, we find realistic ranges with *m_v_* *<* 1 in experiments where cellular methyltransferase activity was experimentally manipulated (44) (see Fig. S3). To explore how hypomethylation can impact competitive outcomes, we allowed virion methylation to range from fully methylated (*m_v_* = 1) to half methylated (*m_v_* = 0.5).

10.1128/mBio.00873-21.3 FIG S3 Conjugal efficiency as a function of endonuclease resistance. The data are redrawn from Elhai et al. (44) with linear regressions and shows the conjugal efficiency of plasmids transferred to *Nostoc* PCC 7120 (formerly *Anabaena*). Several plasmids were generated that varied in the number of restriction sites and tested for conjugal efficiency when the conjugal donor expressed (triangle) or did not express (circles) methyltransferases that correspond to the recognition sequence of a host endonuclease (color). Solid lines represent the regression of unmethylated conjugal transfer efficiency of plasmids, while dashed lines are the methylated conjugal transfer efficiency of plasmids. The asterisk denotes a data point removed from the AvaII unmethylated regression because this is likely the limit of detection and not the true efficiency of transfer. Download FIG S3, TIF file, 3.0 MB. Copyright © 2021 Papoulis et al. 2021 Papoulis et al. https://creativecommons.org/licenses/by/4.0/ This content is distributed under the terms of the Creative Commons Attribution 4.0 International license.

Qualitatively, competitive outcomes in the memory model appear to resemble a mix of general and parallel model outcomes (Fig. 2G to I). A low nutrient supply selects monocultures of the competition specialist, and the defense specialist invades with increasing nutrient supply rates (Fig. 2F and I). However, this happens at a much lower supply rate relative to the control models because all viruses carry the methylation pattern of the competition specialist, effectively nullifying its single RM system (Fig. 2F and I). At high density, fitness and coexistence of competitive (*n* = 1 RMPG) and defensive (*n* = 2 RMPG) types was dependent upon parameter selection. In Fig. 2F, the defense specialist is dominant at high nutrient supply and coexists with the competitive type, resembling the parallel model when nearly all virions are fully methylated. However, the spread of the green ribbon in Fig. 3I reflects the heterogeneity of outcome in the memory model due to parameter selection. Importantly, when there was coexistence, the defense specialist dominated the community at high nutrients in nearly every simulation. The source of variation between competitive exclusion and coexistence was the virion methylation parameter. Unlike near perfect methylation of virions that establish parallel-like infections (Fig. 2F, *m_v_* = 0.99), decreasing virion methylation increases the effectiveness of the defense specialists RMs against viral populations and facilitates competitive exclusion at high nutrient supply (Fig. 2I).

When RM escalation or de-escalation is considered, the memory model drastically differs from the predictions of the other two models (Fig. 3C). As in the coculture (Fig. 2), methylation weakens the protective effect of each additional RM, leading to far more modest gains in cell abundance per RM along the nutrient supply gradient (Fig. 3C). Moreover, the degree of virion methylation toggles the gains between a very gradual increase in cell abundance with respect to the nutrient supply rate (Fig. 3D, purple line, *m_v_* = 0.9) or more drastic gains (*m_v_* = 0.75 and 0.5; Fig. 2D), approaching those of the control models (where *m_v_* ≈ 0; Fig. 3D).

### RM identity impacts coexistence between competitive and defensive populations.

**FIG 4:**
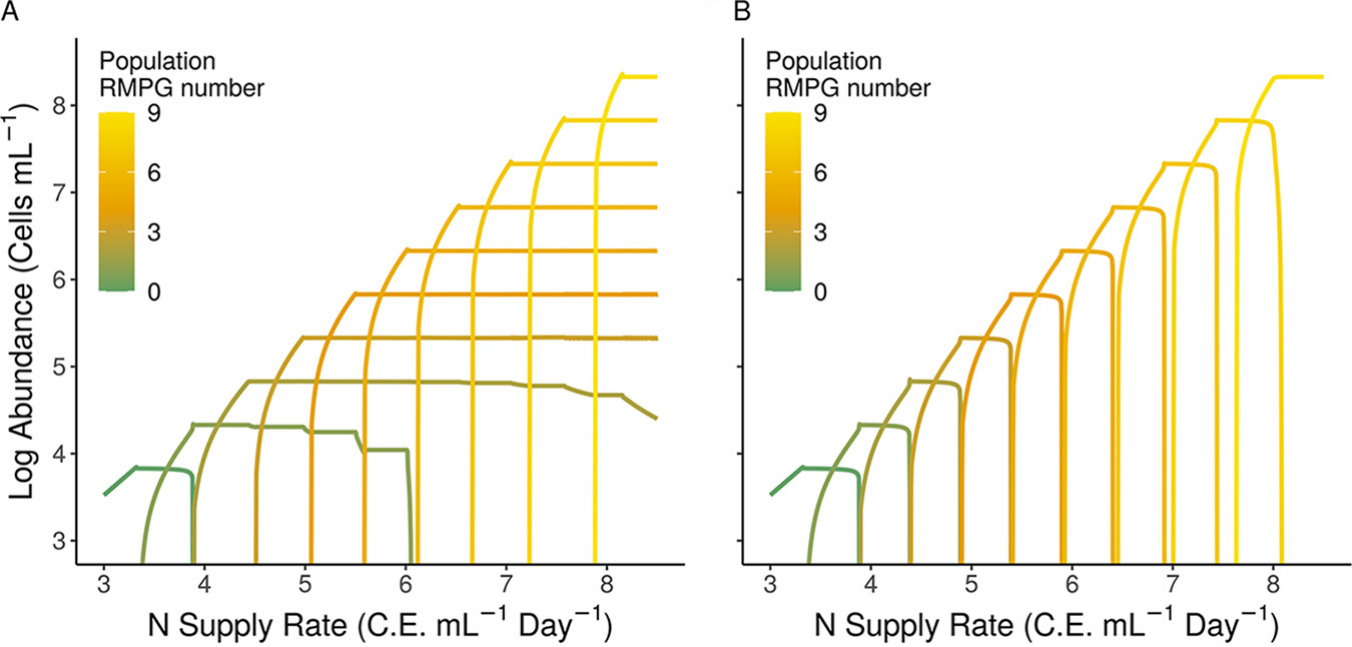
Identity of RM systems among populations determine coexistence of competitive and defensive types in the memory model. (A and B) “Unique set” and “subset” RM communities, respectively, with theoretical methylated viral bursts (*m_v_* = 0.75). C.E., cellular equivalents.

**FIG 5:**
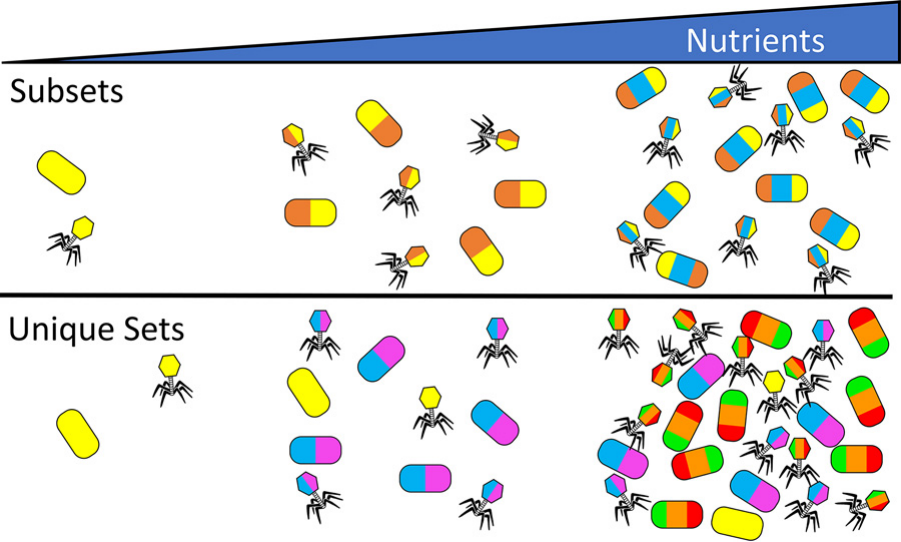
Impact of RM identity on community structure. When populations of prokaryotes compete for resources in the presence of phage, the steady-state community structure depends on nutrient availability and the RM set type. Each color rendered on the bacillus hosts represents a different complete RM system, while colors rendered on phage represent the adopted methylome of the host. The number of bacilli or phage denotes relative abundance at oligotrophic, mesotrophic, and eutrophic conditions along a resource supply gradient. As the environment shifts from an oligotrophic (left) to a eutrophic (right) environment, the optimal number of RMPG increases, with either concomitant competitive exclusion of the low-RM bacilli when RMs exist as subsets or the coexistence of high- and low-RM bacilli when RMs exist as unique sets. Subsetting promotes low RM diversity, whereas unique setting promotes both higher diversity and a higher carrying capacity.

So far, we have assumed that when hosts acquire new RMs, their existing set does not change via loss or divergence. We relaxed the assumption in the memory model that all RMs exist as “subsets” by allowing all populations to have “unique sets” of RMs, representing the endpoint of a diversification scenario where multiple rounds of gene gain and loss have taken place. For both “subset” and “unique set” scenarios, increases in nutrient inflow rate led to numerical dominance by genotypes with progressively higher RMPG (Fig. 4). However, while model communities with RM subsets predict competitive exclusion of cells with fewer RMPG (Fig. 4A and Fig. 5, top), model communities with unique RM sets predict coexistence of nearly all populations (Fig. 4B and Fig. 5, bottom) at sufficiently high nutrient loads.

## DISCUSSION

This study examined the interplay between bottom-up and top-down controls in harmful algal bloom (HAB) ecology, namely, the ability of cyanobacteria to grow and sustain dense populations under constant threat by viral attack in different nutrient contexts. Specifically, we focused on RM defense systems and the possible evolutionary drivers of extensive accumulation of RM defenses in the bloom-forming cyanobacterium *Microcystis*.

Reasoning, as others have (31, 43), that the quantity of RMPG is intrinsic to ecological strategy, we focused our investigation on two ecological extremes established by resource availability: high-density eutrophic systems and low-density oligotrophic systems. Eutrophic systems promote dense blooms of not only *Microcystis* but also *Planktothrix*, *Anabaena*, and *Dolichospermum* in lakes across the planet (34, 38, 49), and consistently, these genera had the highest number of RMPG in cyanobacteria (Fig. 1C). Like *Prochlorococcus*, *Synechococcus* can account for a substantial fraction of the phytoplankton community in the oligotrophic ocean (46), and both genera had below-average RMPG. An intergenus difference in RMPG between oligotrophy and eutrophy could also be observed in intragenus comparisons of different *Synechococcus* isolates, further suggesting that nutrient availability drives pressure to retain RMs. In contrast to intracellular heterotrophic bacteria such as *Wolbachia* or Chlamydia (see Fig. S1 in the supplemental material), the low abundance or complete absence of RMs in these picocyanobacteria cannot be attributed to a lack of phages in their ecosystems, since they play host to a diverse array of viruses, and these viruses are suspected to contribute significantly to mortality of their hosts *in situ* (50, 51).

While our bioinformatics and subsequent modeling focused exclusively on RM defenses, it is important to recognize that RM represents only one form of antiviral defense available to prokaryotes. Prokaryotes can develop resistance to phage through a variety of mechanisms, including the alteration of phage receptors, the production of extracellular matrix, and the production of inhibitors (11, 12, 52). Given the disparity in RMPG between picocyanobacteria and HAB-forming cyanobacteria, we expect other costly defensive mechanisms (e.g., CRISPR and toxin-antitoxin) to also be enriched in eutrophic environments and dispensed in oligotrophic environments over evolutionary time (53), and the observation that prokaryotes with CRISPR-Cas systems have statistically higher RMPG may reflect this reality (32). What remains unclear is whether HABs also occupy the top percentile of other defensive functional groups relative to the rest of the prokaryotic world or if RMs are specifically well suited as defense systems in these species.

Collectively, the bioinformatic results of this work strongly suggested a causal link between increased environmental nutrient availability and the investment in RM antipredator defenses. This relationship is inconsistent with the outcome of a previous modeling study of host receptor mutations and RMs, indicating that RMs are favored at both low and high nutrient availabilities (47). It is also inconsistent with suggestions for terrestrial ecosystems that decreased resources lead to increased investments in defense (54). The reasons for the contradictions are not entirely clear, but in comparing the outcomes between our models and those of the prior RM study, we suspect that previous models assumed that receptor mutants never have a viral predator, and the costs of receptor mutations are higher than that of RM additions. We note that even if resistance from a cell surface mutation is more costly, the resistance would only apply to viruses that exploit that specific cell-surface element to gain entry, and only until a new viral variant arises that can overcome the mutation. Since RMs offer host protection regardless of viral entry, they are an evolutionary parsimonious way to increase viral resistance to all dsDNA viruses in the environment, especially when virus-host contact rates increase in eutrophic conditions.

Our mathematical population models evaluated ecological and molecular mechanisms driving enhanced RMPG in HAB-forming cyanobacteria. Our modeling builds upon prior theoretical (39–43) and experimental (55–57) investigations which have established that the fitness value of antipredator defenses can depend on nutrient supply rate. Underlying this dependency is the assumption that defense benefits come at a cost associated with nutrient utilization ability. We adopted similar approaches in our parallel and general models that served ultimately as controls for the consequences of phage methylation by RMs. While the control models—agnostic to the mechanism of defense—predict nutrients increase the selection for more RMPG, they cannot account for the extent of RMPG escalation evident in genomes of HAB-forming cyanobacteria. This prompted us to consider the special feature of RM defenses, namely, the memory conferred by the host RM system on the progeny phage. While not addressing nutrient influences *per se*, prior studies have reported (24, 47, 48) that memory can affect competition outcomes. When we introduced a memory aspect to our resource gradient model, there was a significant improvement in the ability to capture RM gain and loss events. Like the control models, the memory population model predicts that there is selection for defense against top-down controls with increased environmental nutrient loading, despite assumed costs to resource competitiveness. Unlike the control models, however, the memory model could also explain the high degree of RM accumulation observed in *Microcystis* and other HAB-forming cyanobacteria. Through the mechanism of host methylation of viral progeny, the per-RM increase in fitness is drastically reduced, requiring more RMs to achieve incremental improvements in fitness as nutrients increase.

Our final examination with the memory model evaluated the emergence of communities with differing assumptions about the structure and identity of RM systems. We found that innovation of novel RM sets tends to promote coexistence (Fig. 5). The role of RMs in promoting diversification was modeled in prior work by Sneppen et al. (48), who demonstrated that RM acquisition promotes invasion and that diverse RMs can facilitate long-term coexistence between many bacterial strains. Our results confirm this prior study and builds upon their results by assessing how virion methylation impacts selection for RMPG in contrasting resource environments.

Our modeling suggests in nutrient rich environments, RM diversity is a powerful selective force. This may help explain why RMs are so tightly associated with horizontal transfer (58). Indeed, Oliveira et al. (58) highlighted the diversity of mobile genetic elements RMs are associated with as they are found on plasmids, prophages, transposons, integrative conjugative elements, and integrons. We note that the evolutionary history of the genus *Microcystis* is riddled with indicators of extreme genomic plasticity (59, 60), which could be the result of extreme pressure to innovate viral defense systems, contributing to their rapid genomic turnover (30, 61–64).

### Caveats and future directions.

Our hypothesis connecting RMPG with nutrient loading and viral methylation was the result of our modeling efforts to reconcile the vast number of RMPGs in HAB-forming cyanobacteria with the multiplicative efficacy of RMs in preventing phage attack. For this initial effort our models were necessarily simplified, but we recognize that additional factors will almost certainly contribute to the fitness value of RMPG, and in the following paragraphs we outline some of these factors that should be addressed in future studies.

In a prior study of a smaller set of genomes, Zhao et al. (65) noted a trend that RMPG values were higher in genomes of filamentous compared to unicellular cyanobacteria. In our larger data set, this trend was noticeably less robust, since the filamentous genera *Trichodesmium*, *Pseudanabaena*, *Leptolyngbya*, and *Moorea* or the heterocystous genus *Nostoc* (66) all had low RMPG values. Thus, while cellular organization may play a selective role in RMPG, its role is perhaps not as strong as the role that ecology, specifically nutrient loading, plays.

While the connection between high RMPG and bloom formation in cyanobacteria is striking, it is not universal: *Trichodesmium* forms blooms in marine surface waters (67, 68) and has an RMPG of 2 for the single genome available. This inconsistency may be reconciled by the actual cell densities of blooms. Global satellite monitoring shows that *Trichodesmium* rarely achieve chlorophyll *a* concentration higher than 1 μg liter^−1^, with the majority only showing 0.25 μg liter^−1^ (69). While generally considered blooms, these biomass densities pale in comparison to some HAB-forming prokaryotes with higher RMPG. For example, *Microcystis*, with a mean RMPG of ∼16, blooms in the western basin of Lake Erie or Lake Taihu at 100-fold-higher chlorophyll *a* concentrations (45, 70) than *Trichodesmium*.

In our models, we have assumed that each RM in the genome is active; however, there is reason to suspect this may not always be the case. For instance, some RM systems in Campylobacter jejuni (71) and Neisseria gonorrhoeae (72) are under phase variation control, and similar regulation may occur in cyanobacteria. While methyltransferase activity has been confirmed for some RMs of *Microcystis* (65), the restriction endonuclease components have so far not been verified to our knowledge for any RM. However, even with a conservative estimate that 50% of the RMs in any one *Microcystis* cell are active, our control models still have difficulty explaining RM escalation to this value, whereas the memory model can readily account for this accumulation.

As another caveat, we assumed that there is no redundancy in recognition sequences between RMs gained in our model, that all endonucleases cut at the same rate, and that viruses are assumed to have an equal number of restriction sites. However, natural variation in the number of recognition sequences among wild viruses and the efficiencies of different endonucleases is immense. For example, the endonuclease EcoRV could reduce the number of infections between 0 and 7 orders of magnitude among 21 different phages (20). Future experimental and laboratory work may need to account for possible diversity in cut sites among viruses, and the effects this may have on RM dynamics.

Environment and physiological state can influence RM function in ways that we did not resolve in our modeling, and these influences likely effect RM types differently. For example, Pleška et al. (73) found that there was increased fitness cost of type II RMs in minimal medium compared to rich medium, suggesting that hypomethylation can occur more frequently under these conditions. In bacterial hosts, hypomethylation of restriction sites in the chromosome can lead to DNA cleavage and induction of the SOS response and is likely a major contributor to cellular cost of carrying RMs (73). As reasoned by Pleška et al. (73), hypomethylation may be caused by stochastic gene expression (74) or protein partitioning at the cell level (75). This threat to chromosome integrity would explain why hosts avoid restriction sites in their genome (76). Interestingly, avoidance was more intense for type II RMs than type IIG RMs, suggesting intrinsic cost differences between the different RM types resulting from hypomethylation. These observations imply RM costs could be modeled as a function of the number of host restriction sites, and the cost per site increases due to poor nutrient quality is likely higher when endonuclease and methyltransferase activities are independent, as in type II RMs. One unexplored possibility is that increased costs, resulting from increased likelihood of host genomic hypomethylation, may translate into increased protection against viruses: viruses replicating within hosts may likewise experience hypomethylation from host methyltransferases (77, 78), especially fast-replicating viruses that may fail to fully adopt host methylation prior to encapsidation.

A novel feature of our modeling was the explicit assessment of viral methylation. However, the extent to which viral methylation varies in the natural world is not well understood. Most early studies relied on plaque assays for calculating efficiency: PFU values for initial infections were compared to those for progeny phage infecting their immediate hosts; when initial PFU matched the PFU of the progeny phage, the efficiency of plating (EOP) was declared to equal 1 (23, 25). Since plaque assays cannot quantify noninfectious virions, a significant number of progeny virions could be unmethylated, even in the case of progeny phage infecting their immediate hosts. In only one of these studies was the virus measured by both particle counts and viable counts, and here the difference in counts varied from 0.31 to 0.92 (79), suggesting memory may be less than perfect. Our modeling highlights the potential importance of viral methylation on the occurrence and activity of RMs in natural conditions and points to the need for further experimental investigation to provide constraint on these parameters.

As a final consideration, it will be important to explore the interplay between RMs and CRISPR, receptor modification, and other defense innovations in contrasting environmental conditions. In some cases, such as CRISPR and RM, different mechanisms of defense can function in tandem to increase survival of the population (80, 81); however, it has also been noted that interference between mechanisms can also affect the fitness of defenses: acquiring a receptor mutation might render the intracellular RM or CRISPR defense unnecessary (27, 82). We encourage future studies assessing the complex interplay between mechanisms of defense in nutrient-rich environments and a new antiviral focus to research into the persistence of HABs.

## MATERIALS AND METHODS

### Bioinformatic search strategy.

Because of the diversity in both genomic and domain architecture of RMs, we chose a strategy that uses both BLAST 2.7.1+ (83) and HMMER 3.1b2 (84) to generate alignments to our reference database and then refined our results by using genomic context. Protein profiles are built from hidden Markov models (HMMs) and allow us to identify putative methyltransferases or endonucleases by searching for the specific functional motifs in proteins. By using profiles, we could explicitly detect functional motifs within proteins regardless of the domain architecture, a problem local alignment algorithms like BLAST cannot resolve unless there is a protein with an identical architecture capable of generating full alignments. To ensure we were not aligning multiple profiles to the same residues in each protein, we “competed” profiles that align to 75% of the same residues in a protein and select the profile with the lower E value. We used hmmscan with gathering cutoffs to collect all Pfam (release 31) HMMs (85) that represent experimentally characterized “Gold Standard” methyltransferases and endonucleases found in New England Biolabs’ REBASE (see Data Set S1, Table 3, in the supplemental material) (21). We finalized our reference HMMs after manual curation (see Fig. S4 to S6 and Data Set S1, Table 4, in the supplemental material). In curation, we found ResIII (PF04851) domains were repeatedly observed in various helicases and transcriptional regulators. Since ResIII was common in type I, type IIG, and type III RMs, we retained this domain in our reference HMMs, but added HMMs that would covary with ResIII when a protein was a not part of a RM system to flag false positives (see Data Set S1, Table 5, in the supplemental material). The Gold Standard data set from REBASE contained several endonucleases and a few methyltransferases that did not contain any HMMs from Pfam. As a way to still utilize these sequences to find homologs, all Gold Standard proteins without any identifiable Pfam HMMs were used in BLAST searches with the previously described protein profile searches to maximize our ability to identify RMs in prokaryotic genomes (see Data Set S1, Table 6, in the supplemental material). BLAST alignments were considered a match if the total alignment length was 75% of the query length and the E value was ≤1 × 10^−5^. Once we generated alignments with both HMMER and BLAST, we used genomic context to count the number of full RMs. An RM system was considered complete if there was an endonuclease ≤4,000 bp away from a methyltransferase or if both motifs were detected in one peptide.

10.1128/mBio.00873-21.4 FIG S4 Domains in biochemically characterized type I RM systems from New England Biolab’s REBASE. Individual columns represent the frequency of a protein profile within *n* number of proteins. The NA column shows the proportion of proteins that did not have a detectable protein profile. (A) The frequency of domains found is the specificity subunit of type I RM systems. (B) Frequency of domains found in type I methyltransferases. (C) Frequency of domains found in type I endonucleases. (D) Frequency of domains found in type I RM systems that had subunits concatenated together. Download FIG S4, TIF file, 3.0 MB. Copyright © 2021 Papoulis et al. 2021 Papoulis et al. https://creativecommons.org/licenses/by/4.0/ This content is distributed under the terms of the Creative Commons Attribution 4.0 International license.

Large proteins (>750 amino acids) that contained a methyltransferase domain but did not show any additional motifs to indicate endonuclease activity were subjected to more sensitive search algorithms that are part of the HHsearch suite (86) to evaluate whether they were type IIG RMs since protein size alone can discriminate between type IIG RM and other methyltransferases (see Fig. S6D). We first preclustered these putative type IIG RMs using psi-cd-hit (87, 88) with a clustering threshold of 35% sequence identity and an alignment that covers at least 85% of each protein (parameters: -c 0.35 -aL 0.85 -aS.85 -g 1). Once the clusters were defined, representatives from each cluster were used to build profiles for HHblits. Clusters were considered type IIG proteins if the representative sequence aligned to 3S1S (89), 4PXG (90), 4XQK (91), 4ZCF (92), 5FFJ (93), or 5HR4 (94). Three iterations were used to build multiple sequence alignments with mact = 0.35. Parameters for hhsearch are as follows: *P* = 20, Z = 250, loc, z = 1, b = 1, B = 250, ssm = 2, sc = 1, seq = 1, norealign, maxres = 32000, contxt = context_data.crf.

10.1128/mBio.00873-21.5 FIG S5 Domains in biochemically characterized type II RM systems from New England Biolab’s REBASE. Individual columns represent the frequency of a protein profile within *n* number of proteins. The NA column shows the proportion of proteins that did not have a detectable protein profile. (A) The frequency of domains found is the specificity subunit of type II RM systems. (B) Frequency of domains found in type II methyltransferases. (C) Frequency of domains found in type II endonucleases. Note that the largest column is NA, indicating that most of these proteins require alignments via BLAST. (D) Frequency of domains found in type IIG RM systems. Download FIG S5, TIF file, 3.0 MB. Copyright © 2021 Papoulis et al. 2021 Papoulis et al. https://creativecommons.org/licenses/by/4.0/ This content is distributed under the terms of the Creative Commons Attribution 4.0 International license.

10.1128/mBio.00873-21.6 FIG S6 Domains in biochemically characterized type III RM, type IV RM, and all type methyltransferase size comparison. (A to C) Individual columns represent the frequency of a protein profile within n number of proteins. The NA column shows the proportion of proteins that did not have a detectable protein profile. (A) Frequency of domains found in type III methyltransferases. (B) Frequency of domains found in type III endonucleases. (C) Frequency of domains found in type IV endonucleases. (D) Methyltransferases from New England Biolab’s REBASE were plotted to evaluate the size distributions. We see that the minimum size for most of the type IIG RM systems is 750 amino acids at the dashed black line. Download FIG S6, TIF file, 3.0 MB. Copyright © 2021 Papoulis et al. 2021 Papoulis et al. https://creativecommons.org/licenses/by/4.0/ This content is distributed under the terms of the Creative Commons Attribution 4.0 International license.

This more sensitive analysis revealed that 27,147 of the original 33,633 flagged proteins could be aligned to verified type IIG RMs in the protein data bank. While we believe there is a high likelihood these are RMs, we wanted to determine whether our initial findings (Fig. 1) depend on the veracity of our type IIG calls. When we reanalyzed our RM collection without these putative type IIG RMs, the RM distribution was qualitatively the same: *Planktothrix*, *Microcystis*, *Nodularia*, and *Anabaena* still dominated the tail end of the distribution (see Fig. S2 in the supplemental material).

### Virus-host interaction models.

To model the effects of resource on the selection for defense, we competed theoretical prokaryotic populations for a single resource in the presence of phage. Modeled prokaryotic populations differ only in the number of defense systems (i.e., RMs) they carry, where defensive types have more RMs, and thus greater resistance to phage, relative to competitive types that have fewer RMs. We further assumed a trade-off: investment in RM increases the resistance of the defense specialist to phage, but this comes at a cost to nutrient utilization (95).

We explored the influence of this trade-off on competitive outcomes within three hypothetical system structures with contrasting representations of virus-host interaction (Fig. 2A to C). In the general interaction model (Fig. 2A), the competition and defense specialists are infected by the same phage. In the parallel model (Fig. 2B), the competition and defense specialists are infected by phage that do not cross-infect the other host. In both of these first two models, the viral defense is generic, and RMs could be substituted with phage receptor modification, CRISPR, etc. We emphasize that these models are akin to controls and are meant to establish a simplified understanding of the impact of varied nutrient supply rate to host/virus biomass. In contrast, the memory model (Fig. 2C) is a variation of the control models that accounts for the unique feature of RM defenses: the “memory” bestowed upon surviving phage by the methyltransferase component of the RM defense system in the defense specialist, which renders the phage resistant to the restriction endonuclease (18–20, 23).

Our simulations were completed in a newly developed ODElib Python module (https://github.com/SEpapoulis/ODElib), which aims to make analysis of ecosystem ODE models more tractable by integrating several modules available in the Python ecosystem. We explicitly indicate ODElib dependencies when used for a specific computational task.

All population model simulations were computed using SciPy’s integrate module (96), and all data were managed using Pandas (97). All simulations launched with different fixed nutrient supply rates were allowed to reach an equilibrium steady state. Initial conditions were selected from the previous steady-state values when performing simulations at a range of nutrient supply rates; however, equilibrium values are independent of initial values (see Fig. S7). The final abundances of each population were plotted against the simulation resource inflow to show system state changes in R using ggplot2. A Latin Hypercube Sampling (LHS) scheme (98) from pyDOE2 module was used to randomly pick model parameters over uniform distributions in specified ranges, except for the host resistance per endonuclease (*r_e_*) and baseline infection rate (ϕ), which were drawn from log-uniform distributions (see Table S1 in the supplemental material). LHS is favorable over brute-force random sampling because previous samples are used to make intelligent draws for the next sample, ensuring that random draws are representative of parameter variation in multidimensional space.

10.1128/mBio.00873-21.7 FIG S7 Representative time-dependent solutions. All parameters are identical to those of Fig. 3D to F, except that *S* = 10^6^ C.E. ml^−1^ day^−1^. From left to right, the columns correspond to the general, parallel, and memory models, respectively. From top to bottom, the rows correspond to the initial conditions of 10^2^, 10^4^, and 10^6^ cells ml^−1^ (or virus ml^−1^), respectively. Download FIG S7, TIF file, 1.5 MB. Copyright © 2021 Papoulis et al. 2021 Papoulis et al. https://creativecommons.org/licenses/by/4.0/ This content is distributed under the terms of the Creative Commons Attribution 4.0 International license.

### Model structure.

To explore the selective pressures on bacteria (and other prokaryotes) to increase their defense, we model the competition of *i* bacterial types in the presence of phage. The general model is defined with equations 1 to 4, the parallel model is defined with equations 1, 3, 5, and 6, and the memory model is defined with equation 1 and equations 7 to 9. We will first introduce all parameters and state variables through the definition of the general model and then expand to the parallel and memory models.

All models explicitly define the rate of change of nutrients in the system as follows:

(1) $$\frac{dN}{dt}=S-\overset{n}{\underset{i=1}{\sum }}\alpha \left(1-c\right)B_{i}N$$

where *N* is a nutrient concentration, which is typically measured in molar units. Here, for notational simplicity, we quantify nutrient concentration in “cellular equivalents ml^−1^,” which implicitly assumes a fixed cellular nutrient quota for each competing population. *B_i_* is the *i*th bacterial population (cells ml^−1^) at time *t*. *S* is the nutrient supply rate into the system (cellular equivalents ml^−1^ day^−1^), while α is the nutrient utilization rate (ml cell^−1^ day^−1^), and *c* is the total cost of host defense, implemented as percentage of nutrient utilization lost, and is a function of the set of RMs in bacterial population *i*. We assumed that RMs’ costs are identical and that cost is linear; thus, if one RM system causes *c* to be 0.05, two RMs will cause *c* to be 0.10 for a respective bacterial population and will scale nutrient utilization as a percentage. Moreover, the summation of RM costs cannot be greater than 1. Growth of each bacterial population is defined as:

(2) $$\frac{dB_{i}}{dt}=\alpha \left(1-c\right)B_{i}N-\;\phi \gamma_{i}B_{i}P-\delta_{b}B_{i}$$

where ϕ is the baseline infection rate (virus^−1 ^ml^−1^ day^−1^) of a bacterial population, δ*_b_* is the bacterial loss (day^−1^), and *P* is the abundance of phage time *t*. The vector γ stores coefficients that are calculated based on the number of RMs in each bacterial population and is defined as:

(3) $$\gamma =\left[\begin{array}{ccc} \frac{1}{r_{e}^{{|b}_{1}|}} & \cdots & \frac{1}{r_{e}^{{|b}_{i}|}} \end{array}\right]$$

where *r_e_* is the host resistance conferred by each RM, and *b_i_* is the total number RMs carried by bacterial population *i*; thus, the total defense to phage is the multiplicative protection of all RMs in the general model. For simplicity, we assumed that all RMs in our models have the same protective value. We treat RMs in an organism as a mathematical set which imposes all RMs are unique. Vertical bars denote the cardinality of the set, where cardinality is the total number of elements in *b_i_*. Biologically, we can think of this as each RM system targeting different recognition sequences in DNA. The assumption that RMs are multiplicative is not unfounded, since Arber and Wauters-Willems (20) were able to demonstrate that the defensive value of multiple RMs greatly reduce the efficiency of plating (EOP) of phage on host Escherichia coli strains (22). For example, in these experiments, one RM system decreased the EOP by 1 × 10^−2^, another RM decreased the EOP by 3 × 10^−5^, and together they decreased the EOP of phage to 6 × 10^−7^. Finally, phage replication is determined by the equation:

(4) $$\frac{dP}{dt}=\beta \;\overset{n}{\underset{i=1}{\sum }}\phi \gamma_{i}B_{i}P-\delta_{p}P_{j}$$

where β (virus cell^−1^) is the phage burst size and δ*_p_* is the phage loss (day^−1^). For the parallel model, we alter equations 2 and 4 to accommodate a unique phage population that preys on each bacterial population:

(5) $$\frac{dB_{i}}{dt}=\alpha_{i}\left(1-c_{i}\right)B_{i}N-\;\phi \gamma_{i}B_{i}P_{i}-\delta_{b}B_{i}$$

(6) $$\frac{dP_{i}}{dt}=\beta \phi \gamma_{i}B_{i}P_{i}-\delta_{p}P_{i}$$

In this alternate control model, phage *i* can only infect and reproduce on bacterium *i*. Altogether, equations 1 to 6 represent a diamond food-web ecosystem with predation being “keystone” to maintaining diversity within the ecosystem (39–41), where our general model is a simple diamond food web with a single phage as the predator of competing bacterial populations. Equations 1 to 6 could be extended in an infinite number of ways, for example to include separate compartments for different infection pathways (99) or nonlinear interactions (100). Equations 1 to 6 represent the most transparent and parsimonious model to explore how, to first-order, nutrient supply rate selects number of RMs. Nutrients were explicitly described in cellular equivalence, which is reported as a cell number, to acknowledge that we are remaining agnostic to which nutrient is limiting, and we neglect variability in cell nutrient quotas between organisms. Explicit representation of nonlinear interactions, additional infection pathways, and cellular quotas would introduce additional unconstrained model parameters, while also limiting the clarity with which the mechanisms driving RM selection can be presented. Nevertheless, we also considered an RM-specific ecological virus-host model that allows us to test the effects of viral methylation relative to the general and parallel models, considered control models in this study.

The “memory interaction model” addresses the biological consequences of DNA methylation by RM defenses. Host methyltransferases methylate all DNA indiscriminately, including any replicating viral DNA that evades host defenses long enough to be modified. In this manner, viral progeny adopt the host’s methylation pattern, which confers immunity to the virus when infecting another bacterial cell with the same RM system(s). This adoption of host methylation patterns can be thought of as viral “memory” of its most recent prey. To incorporate the differential infection generated from the modification of viral DNA from host RMs, we create a matrix Γ that stores coefficients between virus-host pairs:

(7) $$\Gamma =\left[\begin{array}{ccc} \gamma_{11}=\frac{1}{r_{e}^{\left|b_{1}-p_{1}\right|+(1-m_{v})|b_{1}\cap p_{1}|}} & \cdots & \gamma_{1j}=\frac{1}{r_{e}^{\left|b_{1}-p_{j}\right|+(1-m_{v})|b_{1}\cap p_{j}|}}\\ \vdots & \ddots & \vdots \\ \gamma_{i1}=\frac{1}{r_{e}^{\left|b_{i}-p_{1}\right|+(1-m_{v})|b_{i}{\cap p}_{1}|}} & \cdots & \gamma_{ij}=\frac{1}{r_{e}^{\left|b_{i}-p_{j}\right|+(1-m_{v})|b_{i}\cap p_{j}|}} \end{array}\right]$$

*b_i_* and *p_j_* denote the RM carried by the host and methylation state of the virus, respectively, and are treated as mathematical sets. Similar to the vector described previously (3), more RMs deteriorate viral resistance to host defenses, but from the cardinality in the difference between *b_i_* and *p_j_* (RM in host *i* and methylation not in virus *j*). The cardinality in the intersection between *b_i_* and *p_j_* (RM in host *i* and methylation in virus *j*) can still reduce viral resistance of phage *j* to bacteria *i*, however, this depends on the virion methylation, *m_v_*. Thus, methylation shared between hosts and viruses cause partial resistance if 0* *< *m_v_ *< 1, with 1 implying perfect methylation of progeny virions, 0 implying a complete lack of methylation, and 0.5 implying half of the restriction sites in the viral genome are methylated.

In reality, each endonuclease has an intrinsic defensive property of defense per unmodified restriction site per cell, represented by the slope of the solid lines from data in Elhai et al. (44) for three endonucleases in *Nostoc* PCC 7120, where we are using conjugal transfer efficiency as an analog for viral resistance (see Fig. S3 in the supplemental material). The positive shift in the slope is due to the reduction in unmodified sites from methyltransferase activity in the host. A slope of <1 suggest there is a small probability for each site to remain unmethylated and is likely an intrinsic property of the methyltransferase and possibly other physiological/environmental constraints (see Discussion). To simplify these cellular processes, we assumed the number of recognition sequences in our theoretical viral genomes to be identical for all RMs and simplified the viral resistance of unmodified and modified states to the idealized parameters, *r_e_* and *m_v_*, respectively. Moreover, we assumed *r_e_* and *m_v_* to be uniform for all RMs, that is, all RM endonucleases have the same efficiency and all RM methyltransferases are equally likely to methylate progeny virions. We considered the sensitivity of our main predictions, allowing inefficient/incomplete methylation due to methylase limitation (77) and unmethylated restriction sites (44). These two observations necessitate that, as long as the methylation of viral progeny is not perfect during replication, viral progenies are susceptible to RM, albeit at a much lower frequency.

To implement the memory model, we must alter the change in both bacterial and phage populations over time so that all phage can infect all bacteria, but each phage population can only emerge from a single bacterial population. This implies that there are an equal number of phage and bacterial types in our system. We accomplished this by using the following equations:

(8) $$\frac{dB_{i}}{dt}=\alpha \left(1-c\right)B_{i}N-\;\overset{n}{\underset{j=1}{\sum }}\phi \gamma_{ij}B_{i}P_{j}-\delta_{b}B_{i}$$

(9) $$\frac{dP_{i}}{dt}=\beta \;\overset{n}{\underset{j=1}{\sum }}\phi \gamma_{ij}B_{i}P_{j}-\delta_{p}P_{j}$$

In equation 8, mortality of bacterium *i* is now due to the collective infections of *j* phage within the environment. In equation 9, the reproduction of phage *i* can only come from bacterial population *i* even though all phage can infect bacterium *i*.

Note that the general model may be thought of as a special case of the memory model. When *m* = 0, we can simplify the exponents in the matrix of equation 7. This simplification leads to equation 7 becoming identical to equation 3 because | *b_i_* – *p_j_* | + | *b_i_* ∩ *p_j_* | is equal to a cardinality of | *b_i_* |. Intuitively, we can think of this as a complete lack of viral methylation which leads to the predicted outcome of the general model.

### Data availability.

All bioinformatic and mathematical modeling work was conducted in executable Jupyter Notebooks, which can be accessed, along with all source code and data, at https://github.com/SEpapoulis/EscalationAndDe-escalationOfRM. ODElib can be found at https://github.com/SEpapoulis/ODElib.
