# Supplementary material for: Nutrient Loading and Viral Memory Drive Accumulation of Restriction Modification Systems in Bloom-Forming Cyanobacteria
Source: mBio. 2021 Jun 1;12(3):e00873-21. doi: 10.1128/mBio.00873-21 (PMC8262939; doi:10.1128/mBio.00873-21)
Supplement: TABLE S1 [file mbio.00873-21-st001.docx]

| Description | Parameter | Units | Figure 3D-F | Figure 3G-I  LHS Sampling Ranges | Figure 4,5  (Unless noted) |
| --- | --- | --- | --- | --- | --- |
| Nutrient Utilization | α | mL cell^-1^  Day^-1^ | 1.0 | Uniform: 0. 5 - 1. 5 | 1.0 |
| Nutrient Supply | S | C.E. mL^-1^ Day^-1^ | Variable; see Figure | Variable; see Figure | Variable; see Figure |
| Burst Size | β | virus cell^-1^ | 25 | Uniform: 5 - 40 | 25 |
| Baseline infection rate | $\phi$ | virus^-1^ mL^-1^ Day^-1^ | 1.78x10^-6^ | Log Uniform: 1x10^-4.75^ - 1x10^-6.75^ | 1.78x10^-6^ |
| Cost of Defense | c | dimensionless | 0.25 | Uniform: 0 - 0.4999 | 0.1 |
| Resistance per endonuclease | $r_{e}$ | dimensionless | 100 | Log Uniform: 10 - 1000 | 100 |
| Bacterial loss | $\delta_{b}$ | Day^-1^ | 0.3 | Uniform: 0.15 – 0.3 | 0.3 |
| Phage loss | $\delta_{p}$ | Day^-1^ | 0.3 | Uniform: 0.15 – 0.3 | 0.3 |
| Virion Methylation | $m_{v}$ | dimensionless | $0.99$ | Uniform: 0.5 – 1 | 0.75 |

Table S1. Parameter Values. Values used for simulations in Figures 3, 4, and 5. Please see methods for a detailed description of equations.
