# Supplementary figures and images for: Nutrient Loading and Viral Memory Drive Accumulation of Restriction Modification Systems in Bloom-Forming Cyanobacteria
Source: mBio. 2021 Jun 1;12(3):e00873-21. doi: 10.1128/mBio.00873-21 (PMC8262939; doi:10.1128/mBio.00873-21)

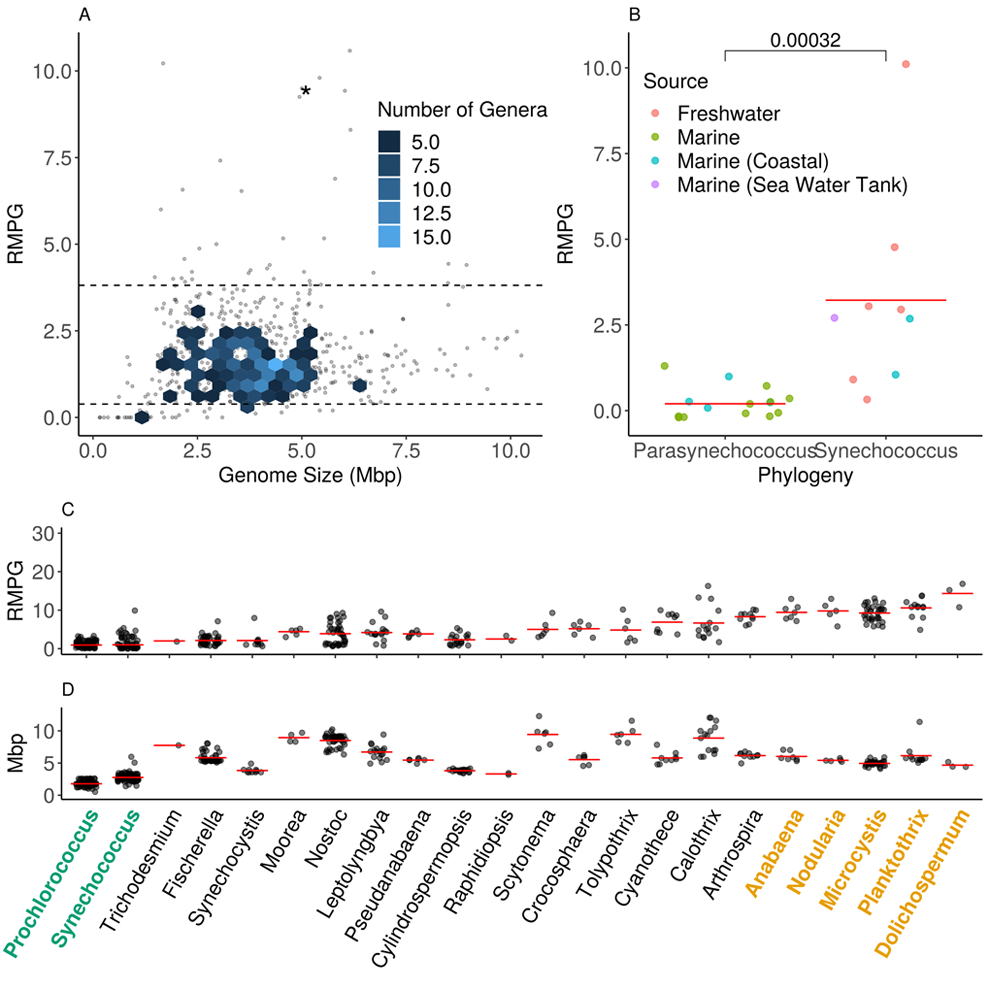

Supplement: FIG S2 [file mbio.00873-21-sf002.tif]

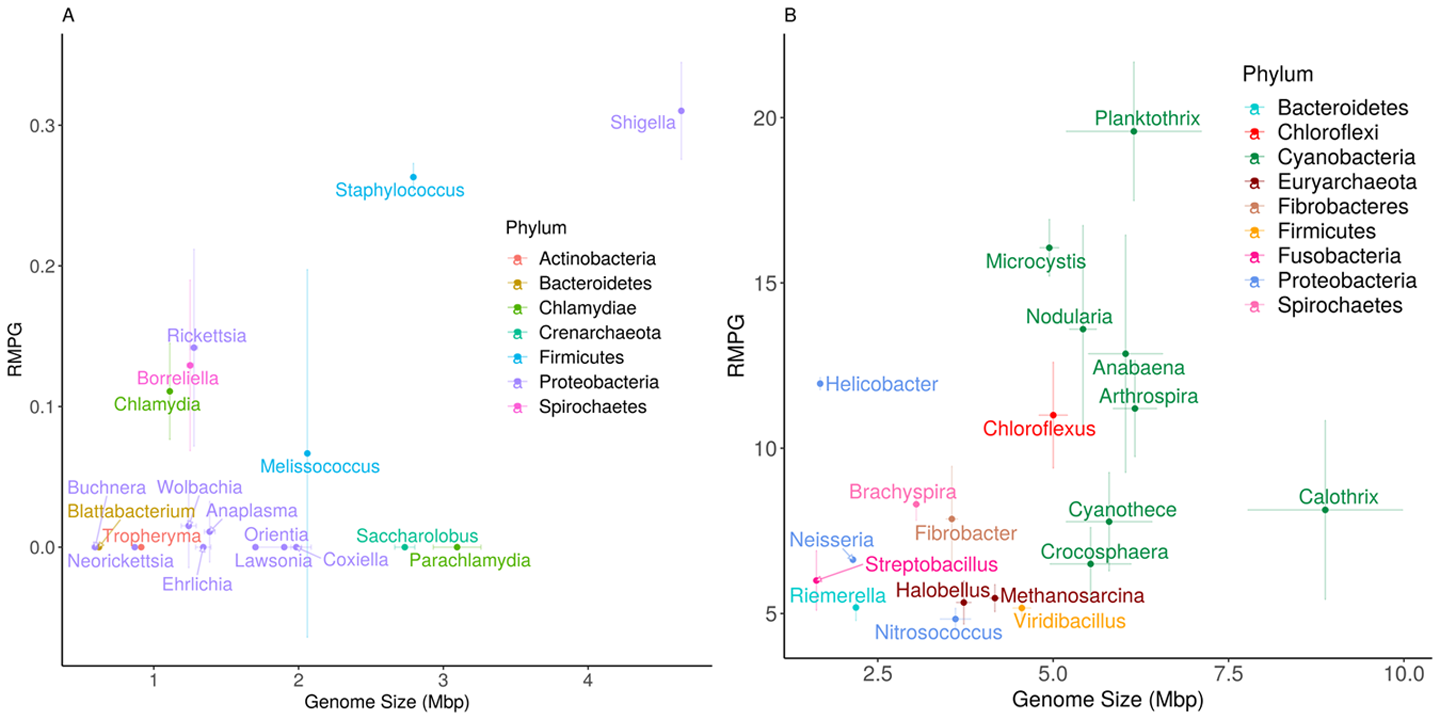

Supplement: FIG S1 [file mbio.00873-21-sf001.tif]

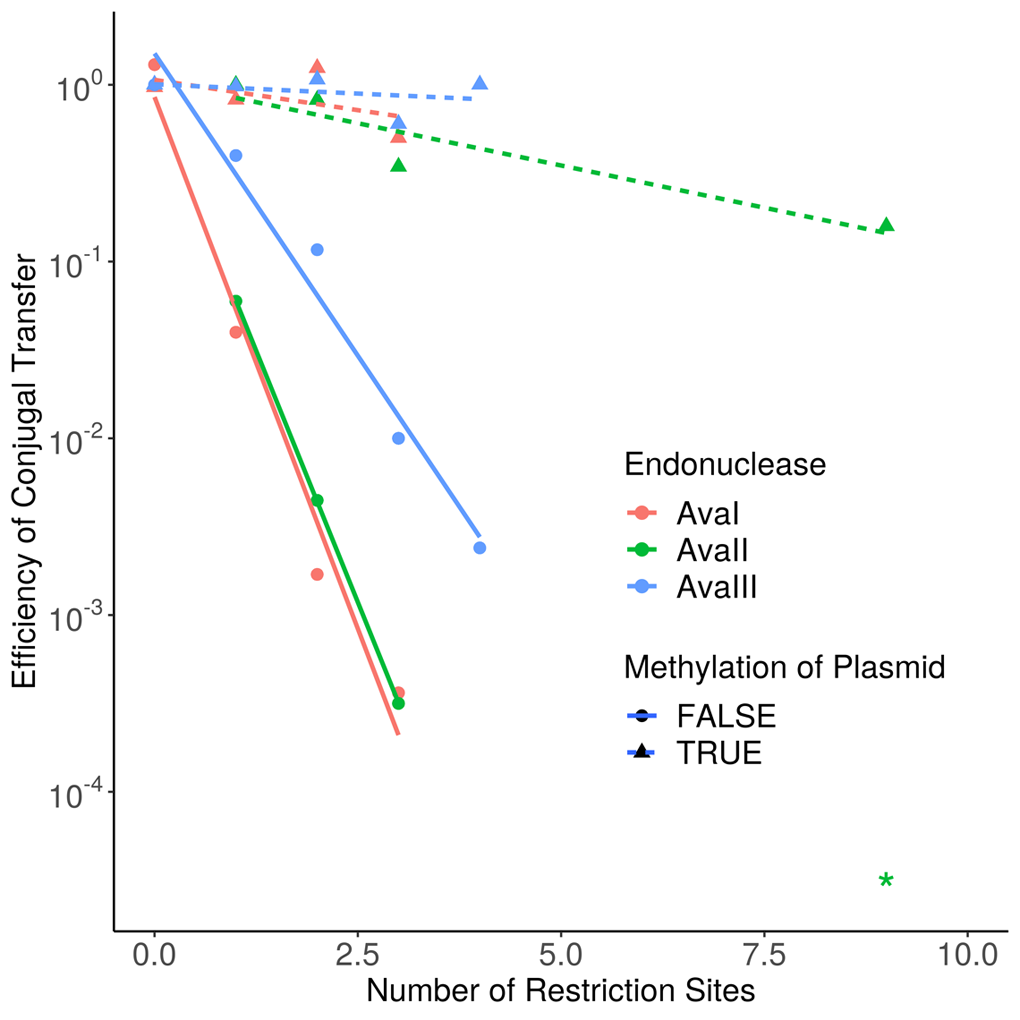

Supplement: FIG S3 [file mbio.00873-21-sf003.tif]

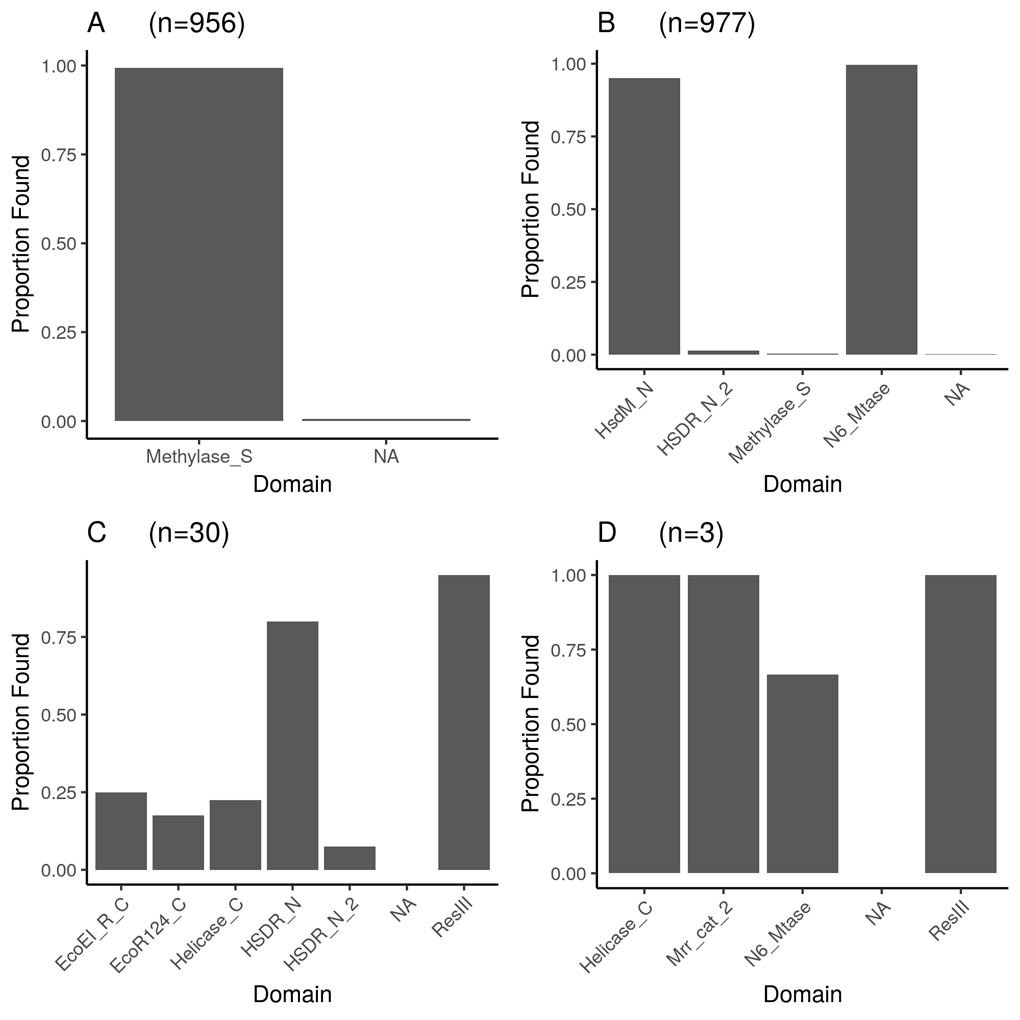

Supplement: FIG S4 [file mbio.00873-21-sf004.tif]

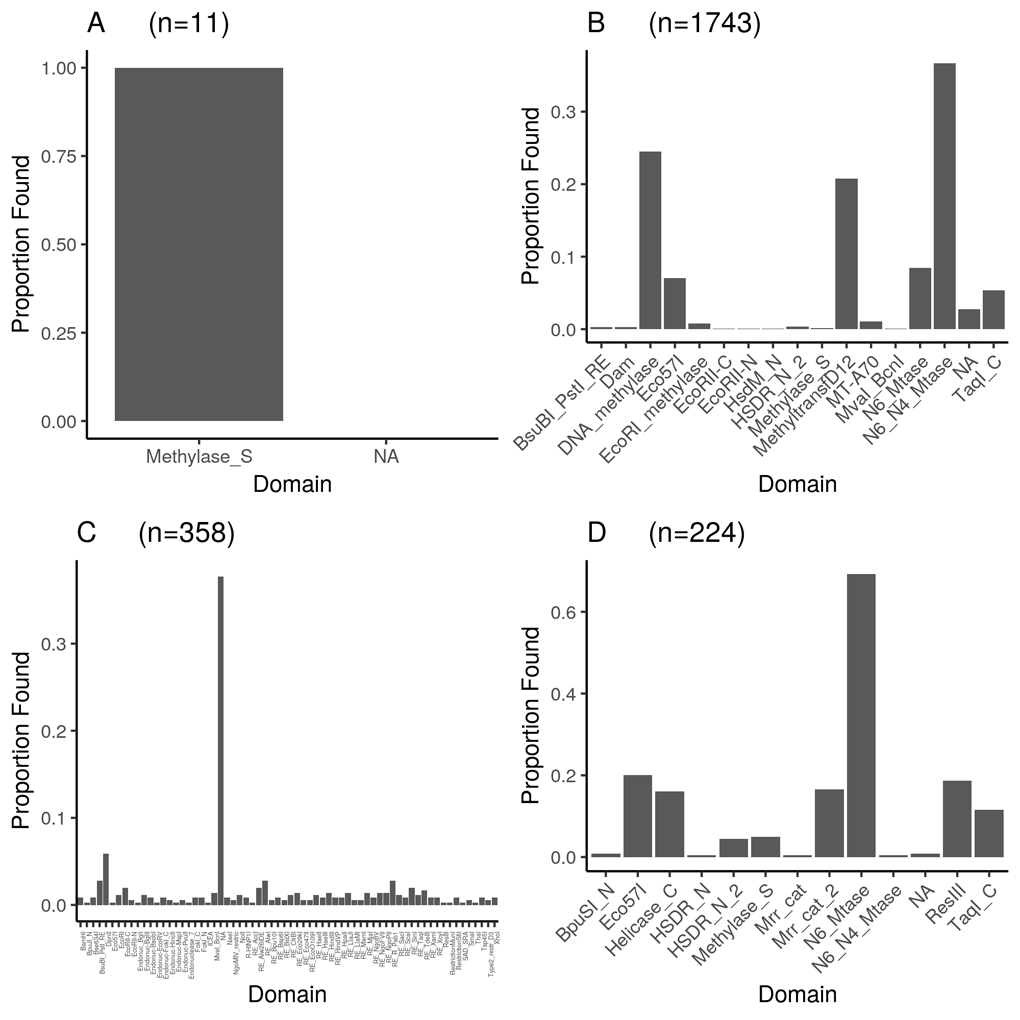

Supplement: FIG S5 [file mbio.00873-21-sf005.tif]

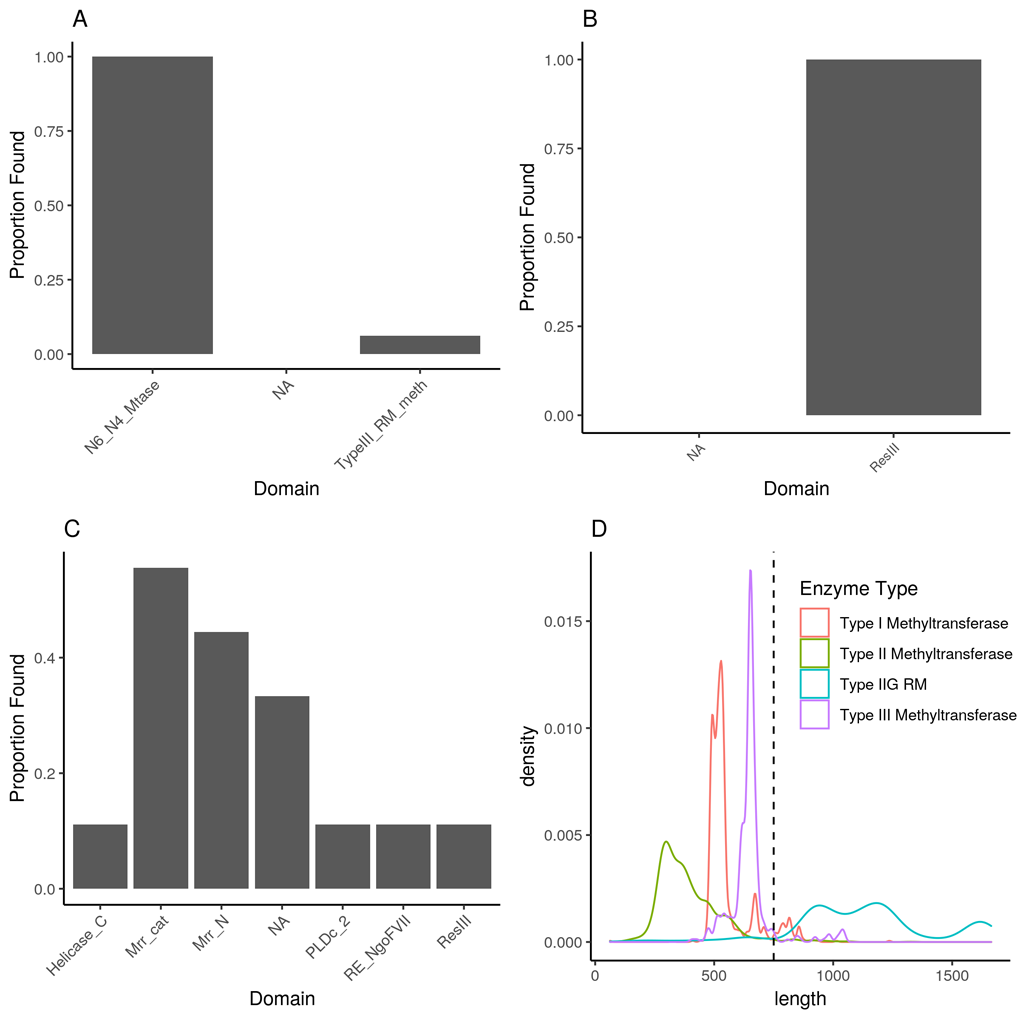

Supplement: FIG S6 [file mbio.00873-21-sf006.tif]
